# The progress and evolutionary direction of menstrual migraine research: a bibliometric analysis from 1994 to 2026

**DOI:** 10.3389/fmed.2026.1817508

**Published:** 2026-06-29

**Authors:** Yixuan Li, Zequn Fu, Xinyun Zhang, Fei He, Canhuan Duan, Si Yuan

**Affiliations:** 1 College of Acupuncture, Massage and Rehabilitation, Hunan University of Chinese Medicine, Changsha, Hunan Province, China; 2 South China Research Center for Acupuncture and Moxibustion, Medical College of Acu-Moxi and Rehabilitation, Guangzhou University of Chinese Medicine, Guangzhou, Guangdong Province, China; 3 School of Traditional Chinese Medicine, Hunan University of Chinese Medicine, Changsha, Hunan Province, China

**Keywords:** bibliometrics, CiteSpace, knowledge mapping, menstrual migraine, research trends

## Abstract

**Background:**

Menstrual migraine (MM) is a subtype of migraine that affects approximately 50–60% of female migraine patients and is closely associated with the menstrual cycle. It is characterized by increased disability, longer attack duration, and more challenging treatment compared with other migraine subtypes. Despite its significant impact on quality of life, the pathophysiological mechanisms of MM are not fully understood, and research in this area is growing rapidly.

**Objective:**

This study aims to provide a comprehensive bibliometric analysis of MM research from 1994 to February 2026, identifying key trends, research hotspots, and influential publications in the field.

**Methods:**

We retrieved articles from the Web of Science Core Collection and PubMed from January 1994 to February 2026. Data analysis and visualization were performed using Microsoft Office Excel 2019, CiteSpace 6.2.R3, and VOSviewer version 1.6.20. The analysis included publication trends, geographical distribution, institutional contributions, author collaboration networks, keyword co-occurrence, and citation analysis.

**Results:**

A total of 1,120 articles were identified. During the study period, the publication volume exhibited a steady upward trend, reaching its peak in 2025 based on the complete annual data from 1994 to 2025. The United States led in publication output (383 articles) and centrality (0.33), followed by Italy (119 articles) and England (82 articles). The University of London (33 articles) and Jefferson University (31 articles) were the most prolific institutions. The most cited authors included Silberstein SD, MacGregor EA, and Stewart WF. Key research topics included “headache,” “prevalence,” “menstrual migraine,” and “women,” with emerging keywords such as “episodic migraine” and “sex differences” indicating current research hotspots.

**Conclusion:**

This bibliometric analysis highlights the growing interest in MM research over the past three decades. The findings reveal key trends, such as increasing publication output, dominant contributions from specific countries and institutions, and evolving research hotspots. Future research should focus on elucidating the pathophysiological mechanisms of MM and exploring more effective treatment strategies. Strengthening international collaboration and interdisciplinary research is recommended to further advance the field.

## 1 Introduction

Menstrual migraine (MM) affects approximately 50–60% of female migraine patients, with women experiencing a 3-fold higher incidence than men (1, 2). It is a type of migraine closely related to the menstrual cycle, characterized by attacks occurring during or around menstruation. These attacks are typically more disabling, longer-lasting, and more challenging to treat (3). The precise pathophysiological mechanisms underlying MM remain incompletely understood, but the pathophysiology is believed to involve estrogen withdrawal and the release of prostaglandins (4). Triptans are commonly used for treatment (1). Because MM is intrinsically tied to ovarian hormone fluctuations—particularly the premenstrual decline in estrogen—it represents a quintessential intersection of neurology and reproductive endocrinology, bridging headache medicine and obstetrics and gynecology (OB/GYN). This condition significantly impacts patients’ quality of life and has become a research focus due to its complex relationship with hormonal fluctuations.

Bibliometrics, also known as scientometrics, refers to a quantitative evaluation of academic publications that measure the scientific progress of a research domain via statistical techniques (5). Therefore, it plays a significant role in depicting the characteristics of a discipline and its future trends. Currently, commonly used bibliometric analysis software includes VOSviewer, CiteSpace, and HistCite (6).

Xiao-Fei Nie, Justin S. Brandt, and others have applied bibliometric techniques to study the field of gynecology, focusing on research trends and geographical disparities (7, 8). Notably, menstrual migraine is a condition that gynecologists frequently encounter in clinical practice, given its direct relationship with the menstrual cycle and hormonal contraceptives. Tingting Lu, Wei, and others have focused on the field of migraine (9, 10), with the former concentrating on identifying research hotspots and the latter on investigating the key role of astrocytes in migraine progression. Despite the significant impact of menstrual migraine, a systematic bibliometric and visual analysis of this field has not yet been conducted. A comprehensive bibliometric study of publications, countries, institutions, journals, authors, and keywords is still necessary.

In this study, we employed bibliometric methods to trace the developmental trajectory of MM research, identify key papers and authors, analyze the evolution of research topics, and reveal citation relationships between different studies. The aim was to uncover research dynamics, assess the impact of research outcomes, and provide data support and directional guidance for future studies. Additionally, we sought to reveal the level of activity and collaborative networks of different countries and regions in MM research, offering new insights and strategies for clinical treatment and scientific research in this area.

## 2 Methods

### 2.1 Data sources

**Figure 1:**
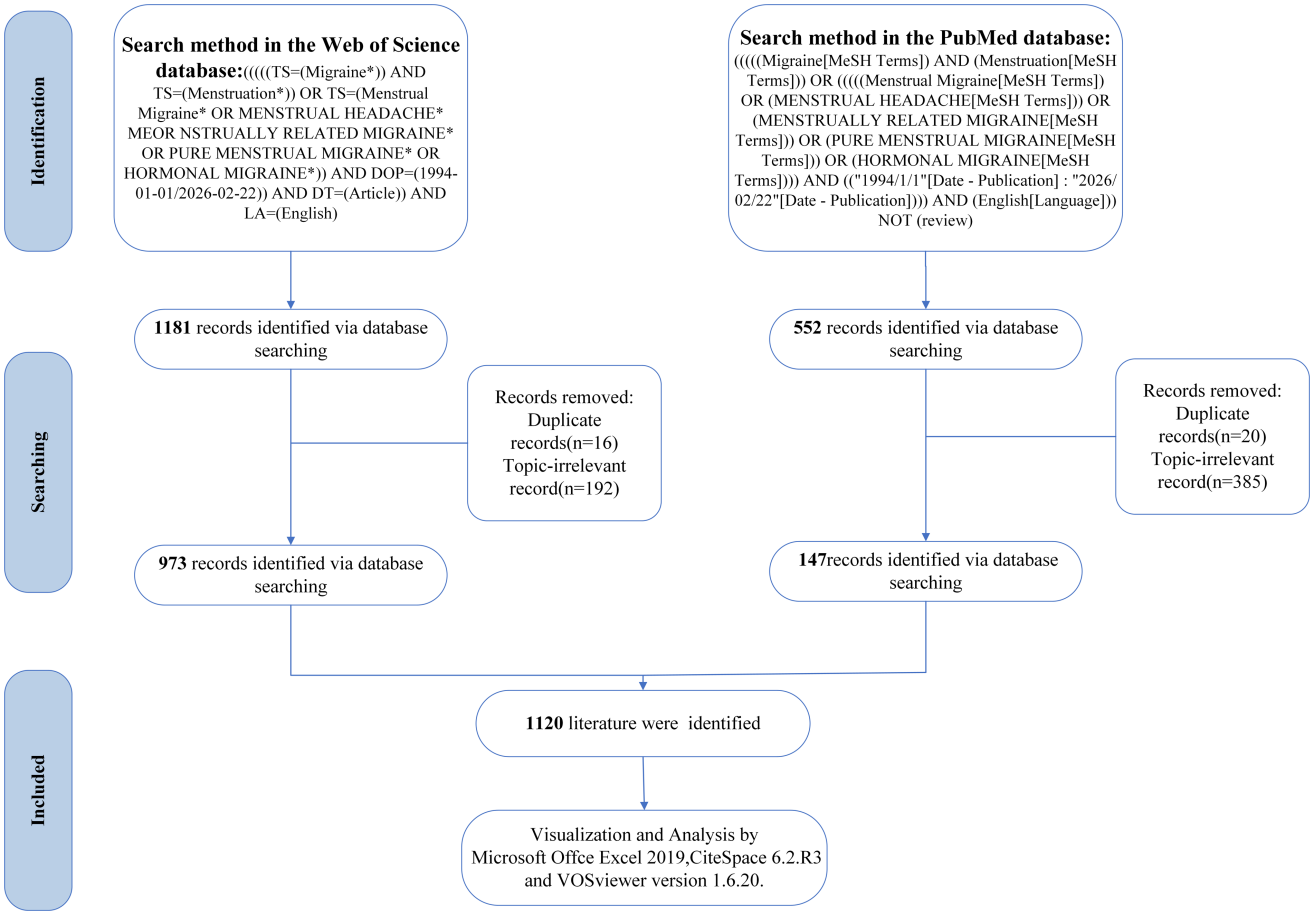
Flowchart of the study.

The literature search was conducted in the Web of Science Core Collection and the PubMed database to identify original articles on menstrual migraine and hormonally related headache disorders. The time span was set from 1 January 1994 to 22 February 2026, and the search was limited to the Article type and the English language. The search was performed on 22 February 2026. The details of the search strategies for each database are provided in Figure 1.

All retrieved records were imported into EndNote, where duplicates were identified through automatic software detection combined with manual verification of titles, authors, journals, and publication years. Screening was then performed by three researchers based on criteria such as titles, abstracts, and keywords. A pilot test on a 5% random sample was conducted before formal screening to standardize the criteria. During formal screening, any disagreement among the three researchers was independently adjudicated by a fourth senior researcher, whose decision served as the final consensus. The inclusion and exclusion criteria were as follows: The study topic must address core aspects, including the pathogenesis, diagnosis, treatment, or prognosis of MM, while studies that merely mention MM without focusing on it as the primary subject were excluded.

After deduplication and exclusion of irrelevant articles, 1,120 unique publications were retained for bibliometric analysis.

### 2.2 Data analysis

In this study, the data analysis and visualization software used were Microsoft Office Excel 2019, CiteSpace 6.2.R3, and VOSviewer version 1.6.20. Microsoft Office Excel 2019 was employed for the statistical analysis of publication trends, data organization, and the creation of relevant tables. CiteSpace 6.2.R3 was utilized to analyze the number of publications listed by country, institution, and author, the intermediary centrality and frequency of keywords, and keyword bursts and to generate visual maps. VOSviewer version 1.6.20 was used to perform journal co-citation analysis.

## 3 Results

### 3.1 Global publication trends

**Figure 2:**
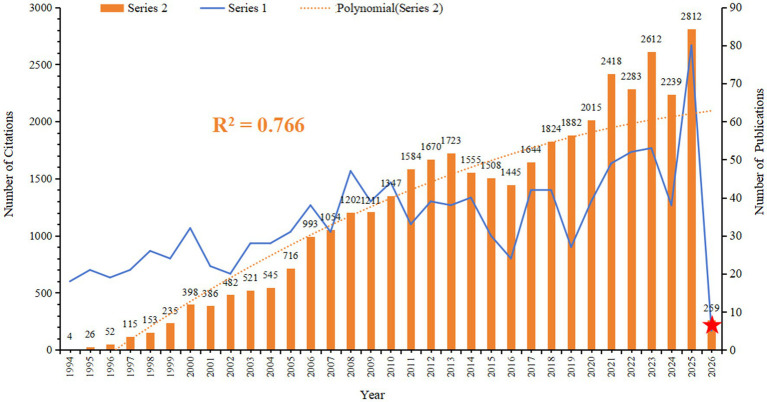
Publishing trend analysis from 1994 to February 2026.

Figure 2 illustrates the annual publication output regarding menstrual migraine (MM) spanning from 1994 to February 2026. Throughout this 30-year duration, the quantity of articles exhibited fluctuations, yet manifested a general upward trajectory, especially subsequent to 2010. From 1994 to the early 2000s, the annual publication count remained at a low level (predominantly below 10), suggesting limited research attention. A gradual increase became apparent around 2005–2010, followed by a more pronounced growth stage after 2015. The highest annual output was observed in recent years (2024–2025). It should be noted that the 2026 data are partial (covering only up to February) and are not comparable to full-year data. A second-order polynomial trend line fitted to the data further confirmed the accelerating augmentation in publications over time. This overall growth pattern is in alignment with the expanding corpus of evidence concerning hormonal mechanisms and targeted therapies for menstrual migraine.

### 3.2 Analysis of countries/regions and institutions

Publications related to the field of menstrual migraine have been authored by researchers from 87 countries. In the network visualization, node size representing a country corresponds to the number of articles it has published in this field. A purple circle is displayed around a node if its centrality exceeds 0.1, indicating a higher level of connectivity within the network. The country co-authorship network consisted of 87 nodes and 162 edges, with a network density of 0.043. This density value indicates a relatively sparse network structure, suggesting that collaborative relationships among countries in this field are selective rather than fully connected, which is common in large-scale bibliometric collaboration networks.

**Figure 3:**
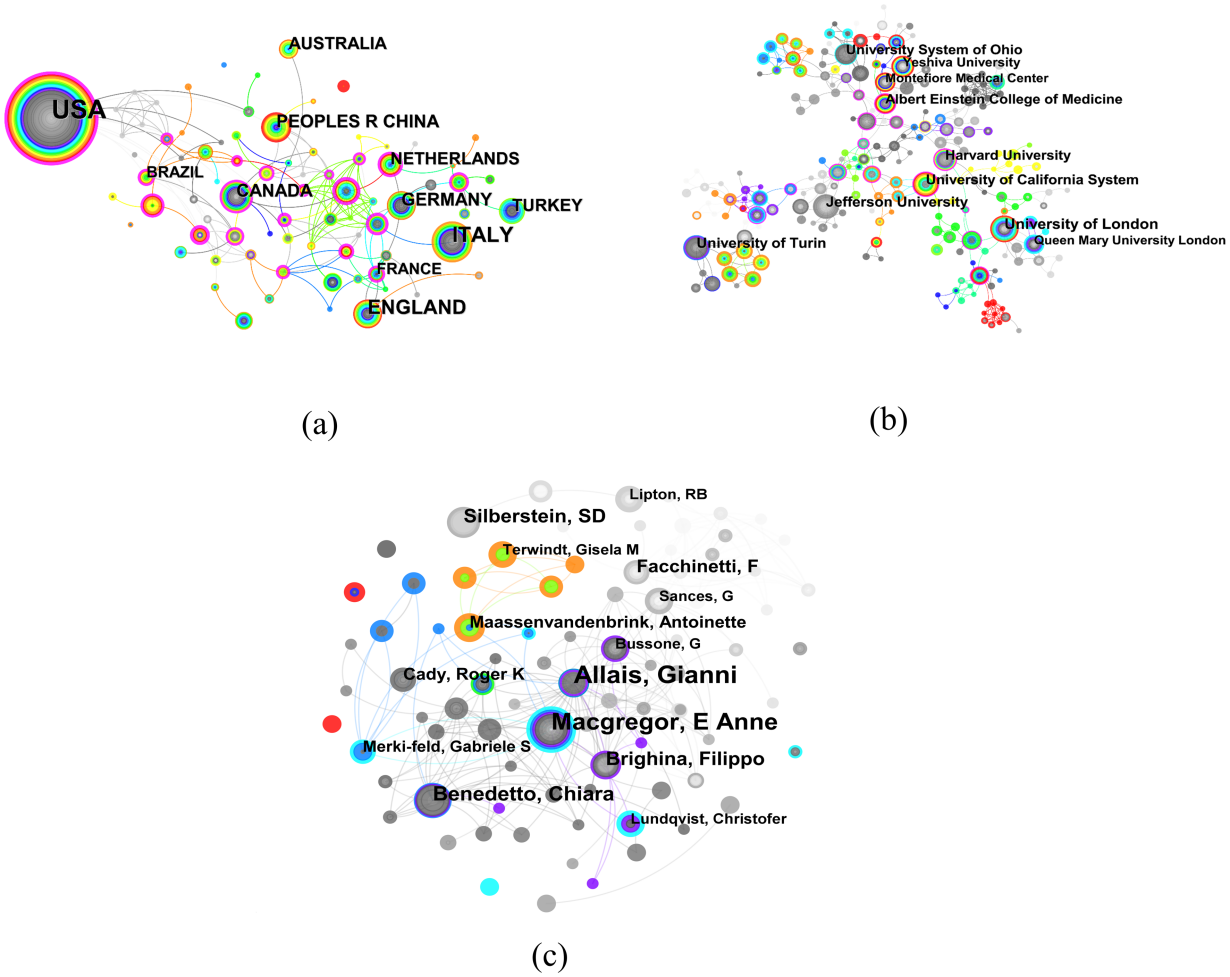
Network analyses in Menstrual Migraine research from 1994 to February 2026. **(a)** Publications and collaborative networks of countries/ regions; **(b)** Publications and collaborative networks of institutions; **(c)** Publications and collaborative networks of authors.

As shown in Figure 3a and Table 1, the top ten countries by publication output were the USA (383 articles), ITALY (119 articles), ENGLAND (82 articles), GERMANY (54 articles), the PEOPLES’ REPUBLIC OF CHINA (53 articles), CANADA (49 articles), TURKEY (43 articles), AUSTRALIA (41 articles), the NETHERLANDS (41 articles), and FRANCE (31 articles). Notably, although Italy has participated in the research collaboration network, its betweenness centrality is 0.00, indicating that it does not serve as a key bridging node between major research clusters. Larger nodes signify a higher volume of publications from that country, while thicker lines between nodes indicate closer collaborative ties. The visualization reveals that the USA not only leads in publication numbers but also maintains strong connections with other countries, exerting significant influence in the field of menstrual migraine. Other countries also exhibit close cooperation, such as between the USA and CANADA, and between ENGLAND and FRANCE. In contrast, despite its high publication output, ITALY has fewer collaborative partners and is not as closely connected with other countries.

**Table 1:** Countries/regions ranked by publications.

Rank	Count	Centrality	Year	Countries
1	383	0.33	1994	USA
2	119	0	1994	ITALY
3	82	0.04	1995	ENGLAND
4	54	0.04	1996	GERMANY
5	53	0.08	1994	PEOPLE’S REPUBLIC OF CHINA
6	49	0.08	1994	CANADA
7	43	0	2002	TURKEY
8	41	0	1994	AUSTRALIA
9	41	0.04	1995	NETHERLANDS
10	31	0.08	1995	FRANCE

The USA believes that the incidence of migraines in women is 3–4 times that in men. After menarche, the incidence of migraines in women increases significantly, which is closely related to the changes in estrogen and progesterone (11, 12). Moreover, there is a focus on studying the relationship between migraines and the menstrual cycle (13), observing the changes in pain intensity before and after menstruation (14, 15), and researching appropriate drugs for treatment, providing new directions for clinical practice (11). The research directions in other countries are similar to those in the USA. However, a study in Italy found that menstruation-related migraines before pregnancy are associated with unimproved headaches in the first and third trimesters of pregnancy (16). A study in England has found that telcagepant does not reduce the monthly frequency of headache attacks, but it does reduce the frequency of perimenstrual headache attacks (15).

The retrieved literature originated from 367 institutions in Figure 3b. The institution co-authorship network comprised of 367 nodes and 494 edges, with a network density of 0.007. The top ten most prolific institutions were distributed across three countries, with seven-tenths of them located in the USA. The top ten universities with the most publications are as follows: University of London (33 papers), Jefferson University (31 papers), University of Turin (28 papers), Harvard University (26 papers), University of California System (25 papers), Albert Einstein College of Medicine (24 papers), University System of Ohio (24 papers), Yeshiva University (19 papers), Queen Mary University London (19 papers), and Montefiore Medical Center (18 papers).

As shown in Table 2, the node representing the collaborative link between the University of Copenhagen and Istanbul University exhibits a betweenness centrality exceeding 1.0 (>1.00). According to the official CiteSpace glossary and Chen (2005) (17), such nodes are defined as “turning points” and are considered critical in network transitions across time slices, indicating a potential structural role in the evolution of the co-authorship network. The main research directions of the top ten institutions in terms of the number of publications are as follows:

**Table 2:** Institutions ranked by publications.

Rank	Count	Centrality	Year	Institutions
1	33	0.07	1997	University of London
2	31	0.07	1995	Jefferson University
3	28	0.02	1997	University of Turin
4	26	0.04	1997	Harvard University
5	25	0.06	2007	University of California System
6	24	0.06	1995	Albert Einstein College of Medicine
7	24	0.02	1996	University System of Ohio
8	19	0.02	1995	Yeshiva University
9	19	0.01	1997	Queen Mary University London
10	18	0.01	1995	Montefiore Medical Center

The University of London (33 papers) focuses its research on the relationship between the rise and fall of estrogen levels during the menstrual cycle and menstrual and menstruation-related migraines (18). It conducts relevant research to evaluate the impact of estrogen supplementation on the onset of menstrual migraines related to estrogen withdrawal during the normal menstrual cycle, the pill-free week of combined oral contraceptives, and the treatment-free week of cyclic estrogen replacement therapy. It also focuses on management strategies for migraines related to different hormonal events. Jefferson University (31 papers) focuses on studying the headache characteristics throughout the menstrual migraine cycle (14), as well as the risks of using the drug frovatriptan during treatment (19, 20). The University of Turin (28 papers) mainly focuses on comparing the performance differences between menstrual migraines and non-menstrual migraines (21), and on the drug treatment of menstrual migraines. Triptans are the gold standard for acute treatment and are commonly used for menstrual migraines. Relevant research has proposed new drug options (such as almotriptan) for menstrual migraines. Institutions such as Harvard University (26 papers), the University of California System (25 papers), and Albert Einstein College of Medicine (24 papers) have similar research directions (Table 3).

**Table 3:** Institutions ranked by Centrality.

Rank	Count	Centrality	Year	Institutions
1	13	0.15	1996	University of Copenhagen
2	14	0.12	2006	Istanbul University
3	26	0.08	1997	Harvard University
4	25	0.08	2007	University of California System
5	33	0.07	1997	University of London
6	31	0.07	1995	Jefferson University
7	12	0.07	1999	Assistance Publique-Hôpitaux de Paris
8	3	0.07	2010	Istanbul Sisli Hamidiye Etfal Training and Research Hospital
9	24	0.06	1996	University System of Ohio
10	24	0.05	1995	Albert Einstein College of Medicine

### 3.3 Analysis of authors

A total of 844 authors participated in the research on menstrual migraines in Figure 3c. The institution co-authorship network consisted of 844 nodes and 1,128 edges, with a network density of 0.003. The top ten authors with the most publications are as follows:

Allais, Gianni (27 papers), Macgregor, E Anne (21 papers), Benedetto, Chiara (14 papers), Brighina, Filippo (12 papers), Silberstein, SD (12 papers), Facchinetti, F (9 papers), Maassenvandenbrink, Antoinette (8 papers), Cady, Roger K (8 papers), Merki-feld, Gabriele S (7 papers), and Lipton, RB (6 papers) in Table 4. Among the top ten authors, only two authors have published more than 20 papers. In addition, we closely observed the cooperation among multiple authors. For example, Allais, Gianni, Benedetto, C, and Benedetto, Chiara have close cooperation; Macgregor, E Anne actively cooperates with Merki-feld, Gabriele S, etc. However, the overall cohesion is not high. The main research directions of the top ten authors in terms of the number of publications are as follows: Macgregor, E Anne (21 papers) conducted relevant research on differentiating between menstrual migraines and non-menstrual migraines (22, 23), laying a certain foundation for providing effective migraine treatment for women. Moreover, through experiments, it was demonstrated that NRP1 may be important in the etiology of MM, providing a basis for understanding how genetic factors contribute to the starting point of the menstrual migraine subtype (24).

**Table 4:** Authors ranked by publications.

Rank	Count	Year	Authors
1	27	1997	Allais, Gianni
2	21	1997	Macgregor, E Anne
3	14	2008	Benedetto, Chiara
4	12	1997	Brighina, Filippo
5	12	1998	Silberstein, SD
6	9	1994	Facchinetti, F
7	8	2017	Maassenvandenbrink, Antoinette
8	8	2007	Cady, Roger K
9	7	2013	Merki-feld, Gabriele S
10	6	1995	Lipton, RB

### 3.4 Analysis of keywords

**Figure 4:**
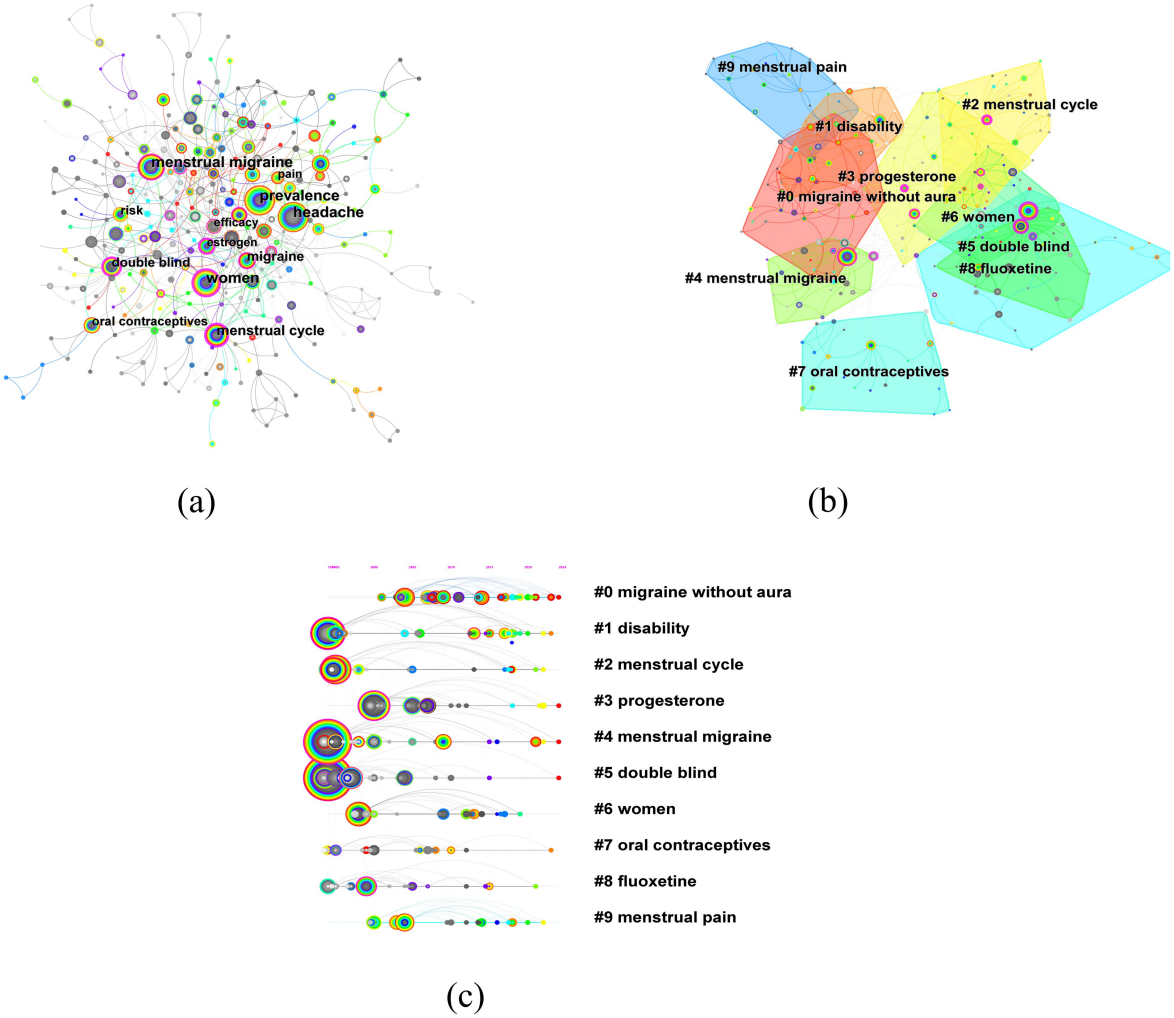
Keyword analyses in Menstrual Migraine research from 1994 to February 2026. **(a)** Co-occurrence keyword network; **(b)** Co-occurrence clustering keyword network; **(c)** Timeline analysis of keywords.

Keyword co-occurrence analysis enables rapid identification of research hotspots within a field. Table 5 Lists the 10 most frequently occurring keywords in menstrual migraine research: headache, prevalence, menstrual migraine, women, menstrual cycle, migraine, double-blind, oral contraceptives, risk, and estrogen. The keyword co-occurrence network comprised 350 nodes and 559 edges with a network density of 0.009. In Figure 4a nodes represent individual keywords. Node size reflects the frequency of each keyword across articles and connecting lines indicate co-occurrence relationships between keywords. “Headache” (*n* = 200) and “prevalence” (*n* = 196) were the most frequent keywords representing principal research directions

**Table 5:** Keywords ranked by publications.

Rank	Count	Centrality	Year	Keywords
1	200	0.05	1994	headache
2	196	0.01	1995	prevalence
3	189	0.1	1994	menstrual migraine
4	187	0.06	1994	women
5	119	0.07	1994	menstrual cycle
6	93	0.09	1995	migraine
7	91	0.15	1998	double blind
8	66	0.02	1998	oral contraceptives
9	62	0.06	1998	risk
10	55	0.13	1999	estrogen

Keywords in Figure 4b were clustered into 10 categories (excluding clusters with <20 articles). The top ten clusters were #0 migraine without aura, #1 disability, #2 menstrual cycle, #3 progesterone, #4 menstrual migraine, #5 double blind, #6 women, #7 women, #8 fluoxetine, and #9 menstrual pain. The clustering solution was validated by a modularity Q of 0.71 (>0.3) and a mean silhouette score of 0.87 (>0.5) both exceeding standard thresholds for bibliometric analysis

The timeline graph illustrates the temporal emergence of clusters and their associated keywords. As shown in Figure 4c: For #0 migraine without aura, the key concept keyword “pain” emerged around 2005. Warfvinge, K, et al. believed that estrogen may regulate the function of trigeminal neurons (25). For #1 disability, the main research keyword “headache” emerged around 1995. Macgregor, EA, et al. proposed the relationship between migraine and changes in estrogen levels, supporting the hypothesis of migraine due to estrogen “withdrawal” in the premenstrual period rather than after ovulation (18). The keyword “cultural adaptation” emerged in 2022. Luo, W, et al. found that the health-related quality of life of patients with menstrual migraine was significantly lower than that of non-menstrual migraine patients in many fields (35). For the #2 menstrual cycle, the main keyword “menstrual cycle” emerged around 1995. Granella, F, et al. considered that in menstrual migraine, perimenstrual attacks lasted longer than menstrual migraine and responded poorly to acute attack treatment (21). The keyword “multiple sclerosis” emerged in 2023. Magdy, R, et al. noticed that female patients with menstrual migraine had more frequent generalized anxiety disorder, panic attacks, and restless leg syndrome than those with non-menstrual migraine (27).

Citation burst refers to a sudden increase in citation frequency over a brief period. It highlights temporal research hotspots and indicates the shift of research focuses over time. It is commonly employed to understand the dynamics and trends of research hot topics. As shown in Table 6, the research phases of menstrual migraine are divided into three periods. Phase 1 (1994–2003) is characterized by an early clinical focus, dominated by terms such as “subcutaneous” (burst: 1999–2004), “sumatriptan” (burst: 2004–2011), “attempted” (1999–2006), “hormones” (2002–2006), and “prophylaxis” (1999–2004), reflecting an initial emphasis on pharmacological interventions and hormonal hypotheses. Phase 2 (2004–2017) is characterized by pathophysiological exploration, with burst terms including “modulation” (2007–2023), “pathophysiology” (2018–2026), “impact” (2018–2026), and “brain” (2018–2026), marking a shift toward understanding the underlying mechanisms of menstrual migraine. Phase 3 (2018–2026) is defined by emerging themes, with persistent bursts for “pain” (2019–2026) and “sex differences” (2021–2026), indicating growing attention to clinical symptoms and gender-specific aspects of the condition.

**Table 6:** Top ten keywords with the strongest citation bursts.

Keywords	Year	Strength	Begin	End	1994–2026
subcutaneous sumatriptan	1999	3.82	1999	2004	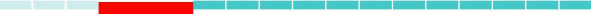
attempted prophylaxis	1999	2.49	1999	2006	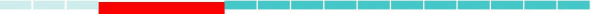
hormones	1999	4.33	2002	2006	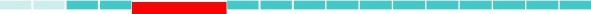
sumatriptan	2004	7.32	2004	2011	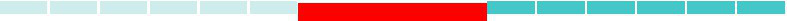
modulation	2017	2.62	2007	2023	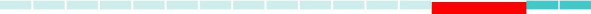
pathophysiology	2018	3.61	2018	2026	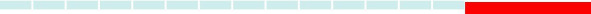
impact	2008	3.15	2018	2026	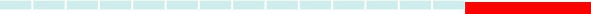
brain	2003	2.91	2018	2026	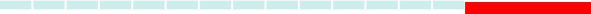
pain	2013	7.88	2019	2026	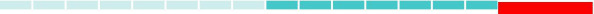
sex differences	2014	6.1	2021	2026	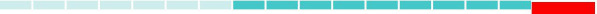

### 3.5 Analysis of cited references

As shown in Table 7, we collected 10 articles that have been cited more than 18 times. Among them, the three most-cited articles are Silberstein SD (2004) (58 citations), Olesen J (2018) (46 citations), and MacGregor EA (2004) (40 citations). They mainly introduce the treatment methods of MM (19, 28). Citation burst in Table 8 refers to a sudden increase in citation frequency within a short period. It highlights the research hotspots in a specific period and indicates the shift of research focuses over time. It can also be used to understand the dynamics and trends of research hotspots. We collected the articles that are still being cited in the field of MM. Among them, the research direction of Olesen J (2018) focuses on the classification of headaches (29).

**Table 7:** Top ten cited references.

Rank	Count	Year	Cited references
1	58	2004	Silberstein SD, 2004, CEPHALALGIA, V24, P2, DOI 10.1111/j.1468-2982.2004.00892.x
2	46	2018	Olesen J, 2018, CEPHALALGIA, V38, P1, DOI 10.1177/0333102417738202
3	40	2004	MacGregor EA, 2004, NEUROLOGY, V63, P351, DOI 10.1212/01.WNL.0000133134.68143.2E
4	34	2004	Granella F, 2004, CEPHALALGIA, V24, P707, DOI 10.1111/j.1468-2982.2004.00741.x
5	30	2013	Bes A, 2013, CEPHALALGIA, V33, P629, DOI 10.1177/0333102413485658
6	25	2006	MacGregor EA, 2006, NEUROLOGY, V67, P2154, DOI 10.1212/01.wnl.0000233888.18228.19
7	24	2004	Silberstein SD, 2004, NEUROLOGY, V63, P261, DOI 10.1212/01.WNL.0000134620.30129.D6
8	23	2006	Brandes JL, 2006, JAMA-J AM MED ASSOC, V295, P1824, DOI 10.1001/jama.295.15.1824
9	23	2003	Couturier EGM, 2003, CEPHALALGIA, V23, P302, DOI 10.1046/j.1468-2982.2003.00516.x
10	21	2000	Stewart WF, 2000, NEUROLOGY, V55, P1517, DOI 10.1212/WNL.55.10.1517

**Table 8:** Emergent analysis of cited literature.

References	Year	Strength	Begin	End	1994–2026
Olesen J, 2018	2018	23.81	2020	2026	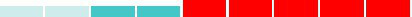
Labastida-Ramírez A, 2019	2019	8.65	2020	2026	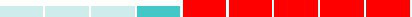
Delaruelle Z, 2018	2018	5.09	2020	2026	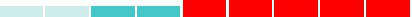
Sacco S, 2018	2018	3.56	2020	2026	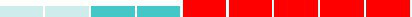
Vetvik KG, 2021	2021	9.38	2021	2026	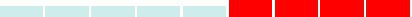
Cupini LM, 2021	2021	5.19	2021	2026	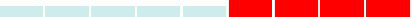
Chauvel V, 2018	2018	3.29	2021	2026	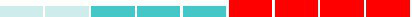
Pavlovic JM, 2020	2020	3.29	2021	2026	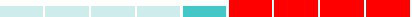
Burch R, 2020	2020	3.59	2022	2026	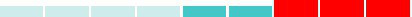

### 3.6 Analysis of cited authors

**Figure 5:**
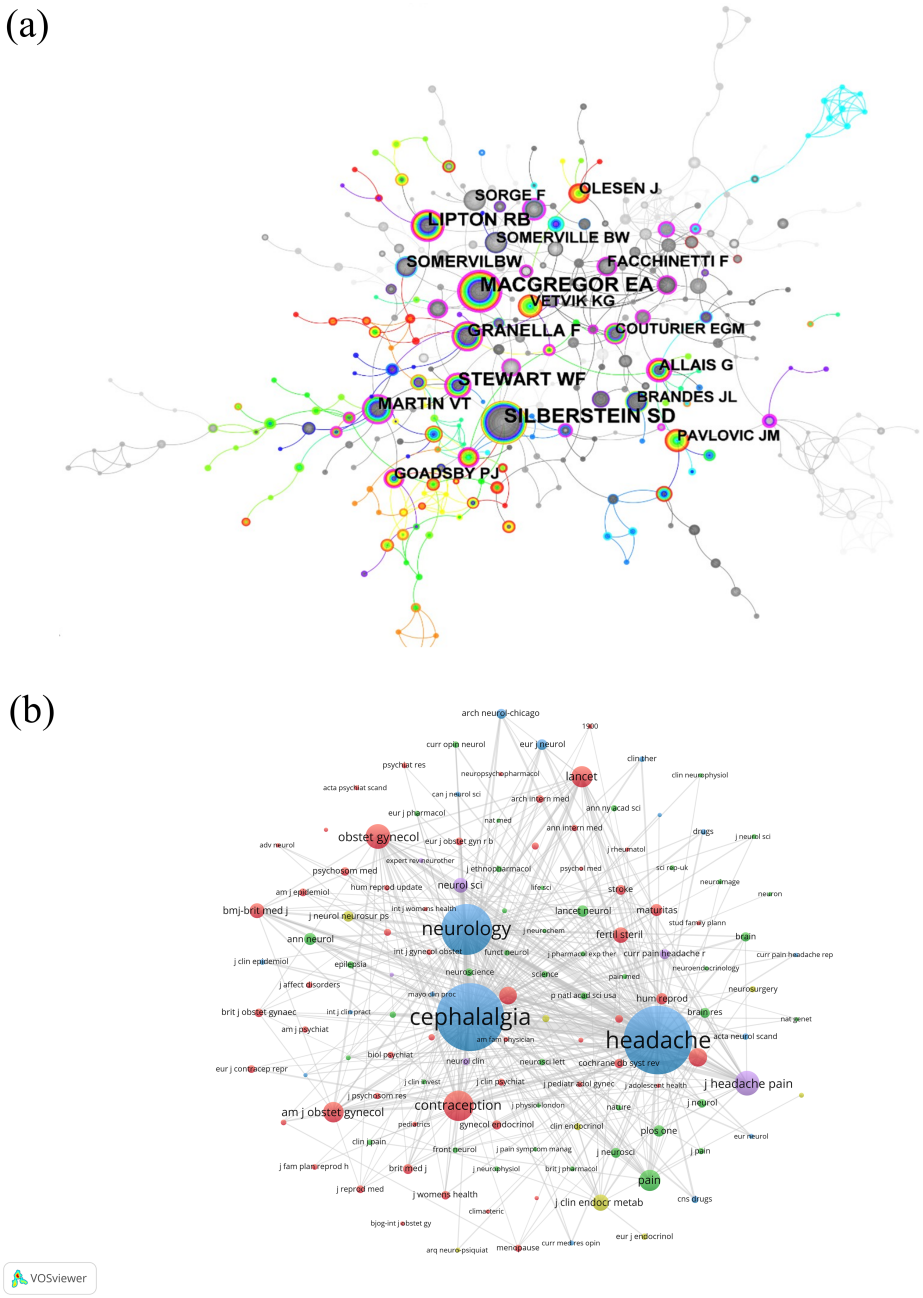
Analysis of cited authors and journals in Menstrual Migraine research from 1994 to February 2026. **(a)** Co-occurrence cited authors network; **(b)** Co-occurrence cited journals network.

The cited author co-authorship network consisted of 367 nodes and 531 edges, with a network density of 0.008. As shown in Figure 5, the top ten authors in terms of the number of cited articles in the MM field are presented as follows: SILBERSTEIN SD (322 times), MACGREGOR EA (292 times), STEWART WF (188 times), LIPTON RB (179 times), GRANELLA F (141 times), MARTIN VT (119 times), SOMERVILBW (97 times), ALLAIS G (89 times), GOADSBY PJ (87 times), and OLESEN J (86 times). Meanwhile, as shown in Table 9, there are close connections among STEWART WF, SILBERSTEIN SD, and MARTIN VT. SILBERSTEIN SD mainly holds that there is a close relationship between menstruation and migraine (30) and explores the possibility of relevant treatments. For example, CGRP-mediated mechanisms have a greater impact on the pathophysiology of menstrual migraine attacks (31).

**Table 9:** Top ten cited authors.

Rank	Count	Centrality	Year	Cited authors
1	322	0.09	1994	SILBERSTEIN SD
2	292	0.28	1997	MACGREGOR EA
3	188	0.31	1995	STEWART WF
4	179	0.12	1998	LIPTON RB
5	141	0.13	1995	GRANDELLA F
6	119	0.28	2005	MARTIN VT
7	97	0.09	1995	SOMERVILLE
8	89	0.2	2005	ALLAIS G
9	87	0.1	1997	GOADSBY PJ
10	86	0.05	2005	OLESEN J

### 3.7 Analysis of cited journals

Figure 5b presents the journal co-citation network constructed using VOSviewer. The network encompasses journals spanning neurology, obstetrics and gynecology, internal medicine, and pain research, underscoring the interdisciplinary nature of MM research. Central nodes, including Headache, Cephalalgia, and Neurology, occupy prominent positions, reflecting their foundational influence. Table 10 lists the ten most-cited journals in MM research. Headache (*n* = 597) and Cephalalgia (*n* = 591) ranked highest, followed by Neurology (*n* = 557). All top ten journals were classified as Q1 in the Journal Citation Reports (JCR), with impact factors ranging from 4.6 to 88.5, underscoring the high academic impact of this field.

**Table 10:** Top ten cited journals.

Rank	Cited journals	Counts	JCR	IF
1	HEADACHE	597	Q1	4
2	CEPHALALGIA	591	Q1	4.6
3	NEUROLOGY	557	Q1	9
4	LANCET	325	Q1	88.5
5	OBSTETRICS and GYNECOLOGY	290	Q1	4.7
6	NEW ENGL J MED	248	Q1	78.5
7	JAMA-J AM MED ASSOC	245	Q1	55
8	AM J OBSTET GYNECOL	230	Q1	8.4
9	J HEADACHE PAIN	224	Q1	7.9
10	PAIN	198	Q1	5.5

## 4 Discussion

### 4.1 General information

This study conducts a comprehensive bibliometric analysis of research on menstrual migraine from 1994 to February 2026. The results demonstrate a clear upward trend in the volume of publications in this field, with the USA leading in both output quantity and centrality, followed by Italy and the United Kingdom. Institutions such as the University of London and Jefferson University have played pivotal roles in advancing this domain. The analysis of keywords and references reveals that the research focus has shifted from prevalence and clinical descriptions to more in-depth themes, such as sex differences, episodic migraine, and hormonal mechanisms. These findings reflect the growing academic interest in menstrual migraine and underscore the necessity of further investigation into its pathophysiological mechanisms and treatment strategies.

From 1994 to 2004, studies suggested that the occurrence of migraine was strongly influenced by hormonal fluctuations in the female reproductive cycle; at least 60% of women affected by migraine associated the periodicity of their attacks with the menstrual cycle (11). From 2005 to 2014, research indicated that triptans could intervene in the pathogenesis of migraine, effectively alleviate associated autonomic nervous system symptoms, and have been widely used to prevent menstrual migraine (12). From 2015 to February 2026, two pathophysiological mechanisms were identified: estrogen withdrawal and prostaglandin release. Although management strategies targeting these mechanisms may be effective, the evidence is not sufficient, and more efforts are needed to understand this condition (4). However, despite these advancements, several unresolved controversies and challenges remain, particularly regarding the role of hormonal fluctuations, treatment efficacy, and the impact on patients’ quality of life.

A central focus of MM research has been the role of hormonal fluctuations, particularly estrogen, in triggering migraine attacks. Estrogen withdrawal during the menstrual cycle is widely recognized as a key factor in MM onset (25). Studies by Silberstein and Lipton have emphasized the dominant role of estrogen in MM pathophysiology (32). However, the involvement of progesterone and other hormones remains contentious. Allais et al. have argued that the interplay between estrogen and progesterone is more complex, suggesting that progesterone may modulate estrogen’s effects (26). This controversy underscores the need for further research to clarify the hormonal mechanisms underlying MM as current evidence is insufficient to establish a unified model.

Silberstein SD (2004) delved into the biological basis of menstrually related migraines, providing insights that aid in accurate diagnosis and help patients identify periods of heightened migraine susceptibility (33). MacGregor EA (2004) further validated the relationship between estrogen fluctuations and migraine attacks, offering a foundation for hormone-related treatment strategies and proposing the potential use of estradiol in future MM therapies (34). These studies underscore the importance of hormonal dynamics in MM but also highlight the complexity of translating these findings into effective clinical interventions.

While clinical trials, particularly double-blind studies, have advanced the evaluation of MM treatments, significant gaps remain in therapeutic strategies. Hormonal therapies, NSAIDs, and emerging targeted therapies show promise, but their long-term efficacy and safety require further validation through large-scale studies. Additionally, cultural adaptation studies have revealed variability in treatment responses across different populations, underscoring the need for personalized approaches (35). The lack of consensus on optimal treatment protocols remains a major challenge as current options often provide only partial relief and fail to address the heterogeneity of MM presentations.

Although recent research has begun to explore the impact of MM on patients’ quality of life, this area remains underdeveloped. MM not only causes physical discomfort but also significantly affects mental health and social functioning. However, standardized tools to assess these impacts, particularly across diverse cultural contexts, are lacking. The 2022 emergence of “cultural adaptation” as a keyword reflects growing recognition of this issue, but more research is needed to develop effective interventions that address the broader psychosocial consequences of MM (35).

The USA has led MM research, with institutions such as Jefferson University, Harvard University, and the University of California System driving advancements in pathophysiology, clinical management, and drug development. These institutions have established extensive international collaborations, further solidifying their leadership. In contrast, countries such as Italy and the UK, while contributing significantly, lag behind in overall output. Italian research, led by the University of Turin, has focused on pharmacological treatments, particularly triptans, while UK studies, such as those from the University of London, have emphasized epidemiological and hormonal aspects (18, 36). This geographic disparity likely reflects differences in research funding, infrastructure, and cultural prioritization of women’s health issues.

Despite the presence of prolific authors such as Macgregor, Allais, and Benedetto, collaboration networks in MM research remain fragmented. While these researchers have made substantial contributions—Macgregor in differentiating MM from non-menstrual migraines (37), and Allais and Benedetto in advancing triptan therapies (26, 38)—the lack of cohesive, interdisciplinary collaboration limits the field’s progress. Strengthening international and cross-disciplinary partnerships is essential to address the complex, multifactorial nature of MM.

### 4.2 Hot spots and frontiers

More recent studies indicate a transition from simplified estrogen-centered explanations toward broader neuroendocrine and cortical network frameworks. The study by Sachs, M. et al. demonstrated that estradiol and progesterone may modulate cortical excitability by potentiating cortical spreading depression and synaptic transmission, suggesting that MM pathophysiology involves dynamic interactions between sex hormones and neuronal signaling pathways rather than isolated hormonal fluctuations alone (39). This emerging mechanistic direction reflects a broader integration of neuroscience, endocrinology, and pain research.

The bibliometric analysis also reveals an important therapeutic transition. Earlier studies emphasized conventional acute headache management, whereas recent research increasingly focuses on individualized and life-stage-specific treatment strategies, including menstrual migraine, menopause-associated migraine, and hormonally induced migraine related to oral contraceptive use. Silberstein S. D. contributed substantially to this transition by integrating hormonal modulation with standard migraine therapies and advocating tailored clinical management approaches (40). The growing prominence of keywords related to personalized medicine and refractory migraine further suggests a shift toward precision-based therapeutic frameworks.

Another notable evolutionary trend is the expansion of research outcomes beyond attack frequency reduction toward broader assessments of disease burden and patient-centered outcomes. Recent studies increasingly address quality of life, psychological comorbidities, functional impairment, and socioeconomic impact, reflecting a transition from purely biomedical models to more comprehensive biopsychosocial perspectives.

Despite these advances, several important gaps persist. Although estrogen withdrawal mechanisms are relatively well established, the roles of progesterone and other neuroendocrine mediators remain controversial. Heterogeneity in diagnostic criteria, study populations, and treatment protocols also limits evidence synthesis across studies. Furthermore, bibliometric results demonstrate persistent geographic imbalance, with research output concentrated in a limited number of countries and institutions.

Collectively, the evolutionary trajectory of MM research appears to be moving toward interdisciplinary, mechanism-driven, and patient-centered approaches. Future progress will likely depend on large-scale longitudinal cohorts, integration of neuroendocrine biomarkers with clinical phenotyping, and strengthened international collaborative networks capable of supporting standardized data sharing and multicenter clinical trials.

### 4.3 Limitations

Although this study has yielded certain insights, it has several methodological limitations. First, the use of only the Web of Science Core Collection and PubMed as data sources may have led to the omission of relevant studies indexed in other databases, such as Scopus or Embase. While Web of Science is commonly used for bibliometric analysis due to its extensive coverage and citation data, and PubMed, as an authoritative database in the biomedical field, ensures the high relevance and professionalism of the included literature in medicine and related disciplines, the exclusion of other databases could still introduce selection bias and affect the generalizability of the research findings. Future studies should consider cross-database searches to enhance the comprehensiveness of literature inclusion.

Second, the citation analysis in this study was based on the retrieved literature corpus, in which even the top-cited reference received only 58 citations. This relatively low citation count may limit the generalizability and statistical power of the citation-based inferences. Future studies with larger datasets and longer time spans are needed to validate and extend our findings.

Finally, the literature search for this study was performed in February 2026; therefore, the data for 2026 are partial, covering only up to February 2026, and do not represent a full year. Although we have marked and presented the partial 2026 data (as of February 2026) with red five-pointed stars in Figure 2 to illustrate recent trends, these data should be interpreted with caution. Readers should note that the publication and citation counts for 2026 may change to some extent once the complete year’s data become available. Future studies should update and validate our findings once complete data for 2026 become available.

## 5 Conclusion

In summary, MM research has advanced considerably over the past three decades. The growing number of studies and expanding knowledge base have elucidated the pathophysiological mechanisms, epidemiological characteristics, and treatment strategies of MM. However, key challenges remain, including achieving a comprehensive understanding of MM pathophysiology and developing more effective treatment strategies. These bibliometric findings underscore the importance of continued research to address these gaps. We recommend strengthening international collaboration, fostering interdisciplinary research, and prioritizing patient quality of life to generate new insights and strategies for the clinical management and scientific investigation of menstrual migraine.

## Data Availability

The original contributions presented in the study are included in the article/supplementary material, further inquiries can be directed to the corresponding author.
